# The intestinal permeability marker FITC-dextran 4kDa should be dosed according to lean body mass in obese mice

**DOI:** 10.1038/s41387-022-00230-2

**Published:** 2023-01-05

**Authors:** Louise M. Voetmann, Bidda Rolin, Rikke K. Kirk, Charles Pyke, Axel K. Hansen

**Affiliations:** 1 Department of Veterinary and Animal Sciences, Faculty of Health and Medical Sciences, University of Copenhagen, 1870 Frederiksberg, Denmark; 2 Global Drug Discovery, Novo Nordisk A/S, 2760 Måløv, Denmark

**Keywords:** Animal disease models, Obesity, Pre-diabetes, Metabolic syndrome

## Abstract

**Aims:**

To investigate the influence of the dose in the FITC-Dextran 4kDa (FD-4) permeability test in an obese mouse model, we tested the bodyweight dose regimen and a lean body mass-based dose regimen in high fat diet (HFD) mice and low fat diet (LFD) mice. We hypothesized that the FD-4 permeation result would be dose-dependent.

**Methods:**

The two dose regimens were compared in HFD and LFD mice. Furthermore, we conducted a dose-response study to test the effect of a low or high dose of FD-4 in weight-stratified lean mice. Gene analysis of tight junctions was also carried out.

**Results:**

The FD-4 intestinal permeability test was dose-dependent as we found a significant increase in plasma levels of FD-4 in obese mice with the bodyweight dose regimen. However, this difference was not detectable with the lean body mass dose regimen, even with variability-adjusted group sizes. However, the qPCR analysis revealed a decrease in tight junction gene expression in obese mice. Furthermore, we found a dose-dependent significant increase in FD-4 measured in plasma samples in lean mice. No significant difference in intestinal weight was observed between lean and obese mice.

**Conclusion:**

Evaluation of the intestinal permeability by FD-4 with the typical bodyweight dose regimen in obese mice will be confounded by the significant difference in dose given when compared to a lean control group. If the test dose is based on lean body mass, no significant difference in intestinal permeability is observed, even with large group sizes. Furthermore, we showed a dose-dependent difference in plasma FD-4 levels in lean mice. Therefore, we conclude that the dose should be based on lean body mass for the FD-4 permeability test if mice with considerable obesity differences are to be compared or to use another test with fixed doses.

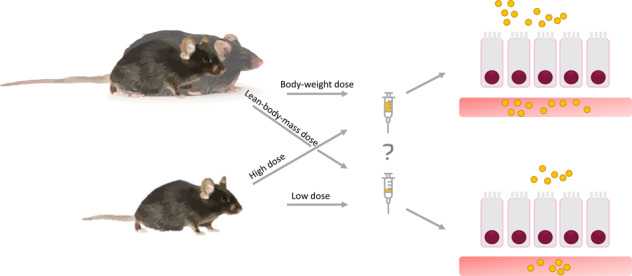

## Introduction

Increased intestinal permeability due to a disrupted intestinal barrier seems to play a vital role in the pathogenesis of obesity and diabetes [1, 2]. In preclinical models of diabetes and obesity, such as the diet-induced obese mice (DIO), this is studied via in vivo permeability tests. A typical test used is FD-4; a method first described by Tagesson and colleagues in 1978 [3]. Years later, it was adapted for studies of diabetes and obesity [4–6]. The FD-4 assay is often used preclinically because of its simplicity, low cost and adaptability in most laboratories. FD-4 resembles the size of a lipopolysaccharide molecule and diffuses passively from the intestine to the circulation via the paracellular route [3, 7]. FD-4 is usually dosed according to body weight (BW) in the intestinal permeability test [4–6, 8, 9]. E.g., in a study by Ahmed and colleagues, the DIO mice gained 130% of their initial weight while the lean mice gained 30%, and the DIO mice received twice the dose of a lean mouse [4]. We hypothesize that a BW-related difference in drug dosages in DIO mouse studies confounds the readout in the FD-4 in vivo intestinal permeability test and that a new dose regimen based on lean body mass (LBM) will remove this dose-dependent difference.

## Methods

### Animal studies

Three consecutive studies were conducted on a total of 45 DIO male C57BL/6 JCrlf mice (Charles River, France) and 70 lean male C57BL/6J mice (Janvier or Charles River, France) aged 8–16 weeks at arrival. Upon arrival, mice were fed either a HFD (D12592, Research Diets Inc.) or a LFD (D12450B, Research Diets Inc.) ad libitum. Mice were acclimatized in minimum 1 week before study initiation. DIO mice were single housed, 2 animals per cage with a partitioning wall between, while the control mice were housed 5 per cage in temperature (22 ± 2 °C) and humidity (50 ± 20 %) controlled rooms with a circadian rhythm (12 h light: 12 h dark; Lights on at 06:00). Mice had ad libitum access to food and water and enrichment in the form of chewing material, climbing racks, and nests provided in the cage. Bedding and enrichment materials were exchanged every week, and animals were inspected by caretakers daily. In the study period, humane endpoints were applied as follows; cases where the animals showed signs of permanent suffering, pain, or fear.

The care and use of mice in these studies were conducted according to national regulations in Denmark and with an experimental license granted from the Danish Ministry of Food, Fisheries, and Agriculture and conducted according to the EU Directive 2010/63/EU on the protection of animals used for scientific purposes.

### Study design

For the studies investigating intestinal permeability in obese and lean mice, mice were weight stratified to the two subgroups, LBM dose or BW dose (lean groups, mean weight 27.2–27.3 g and obese groups mean weight 55,5–55,7 g, *n* = 10 /grp). Mice in the drug-response dose study weighed an average of 29,9 g and were weight stratified to receive a low or high dose of FD-4 (*n* = 10/grp). In the power corrected study (for power calculation, see Supplementary Table 1) using LM dose only, the average weight of DIO mice was 48,2 g (*n* = 25), while it was 28,7 for the lean mice (*n* = 23, two died before study termination). Mice scheduled to LBM dosing were scanned in an EchoMRI Body Composition Analyser (EchoMRI, Houston, TX, USA) the day before study termination. At study end, the mice were 18–24 weeks. On the study day, mice were weighed and fasted from 6 AM to 10 AM before FD-4 (*dissolved* in PBS *at a concentration of 125* *mg/ml)* PO dosing using a stomach tube (for study design, see Supplementary Fig. 1). After retroorbital blood sampling, mice were sacrificed, and the liver and gastrointestinal tract were sampled. All experiments were repeated two to three times and carried out by trained and licensed personnel. Experimenters were not blinded, only at analysis.

### In vivo intestinal permeability test

Mice fasted for four hours before FD-4 (Sigma-Aldrich, USA) were administered orally (600 mg/kg) in a randomized order. Two hours after dosing, mice were anesthetized using isoflurane (induction 5%, maintenance 2% isoflurane, 0.7 L/min N2O, 0.3 L/min O2), and retro-orbital blood sampled in K3-EDTA-coated tubes (Sarsted, Germany) followed by cervical dislocation. The samples were centrifuged (4 °C, 7 min, 8000 g), and plasma was collected in clear Eppendorf tubes (Eppendorf AG, Hamburg). Plasma from PBS dosed mice were used for the standard curve. FD-4 concentrations in plasma were analyzed in duplicates using a spectrophotometer (SpectraMax M4, Molecular Devices, San Jose, CA, USA) with excitation λ 485 nm and emission λ 535 nm.

### qPCR

The jejunum was sampled immediately after termination, snap frozen, and stored at −80 °C for qPCR analysis. All samples were homogenized in 1 ml Trizol (#15596018 Invitrogen), and RNA was extracted using Rneasy mini kit (#74106, Qiagen) with Dnase treatment included (79254, Qiagen) and analyzed by NanoDrop 8000 system (ThermoFisher Scentific). The total amount of RNA isolated was approximately 10 µg per jejunum. Iscript (#1708841 Bio-Rad) was used to prepare cDNA, and the qPCR analysis was performed with TaqmanTM Fast Advanced Master Mix (4444964, Applied Biosystems) and the listed Taqman gene expression assays (see Supplementary Table 2). The genes of interest were normalized to Sdha and presented as fold-change of gene expression (2^–ΔCt^) compared to the lean mouse group. At analysis, the samples were blinded to the analyst.

### Statistical analysis

GraphPad Prism version 9.0.1 (GraphPad Software, San Diego, CA, USA) was used for statistical analysis; *p* values below 0.05 were considered significant. Group comparisons were made using a *t* test or One-way ANOVA. If the data did not show Gaussian distribution by D’Agostino-Pearson test or variances were unequal by the Brown-Forsythe test, the data were log-transformed. Pearson’s correlation analysis was used to test the statistical relationship. Results are shown as mean ± standard deviation.

## Results

### Differences in lean and obese mice were less pronounced when FITC dextran 4kDa was dosed according to lean body mass

**Fig. 1:**
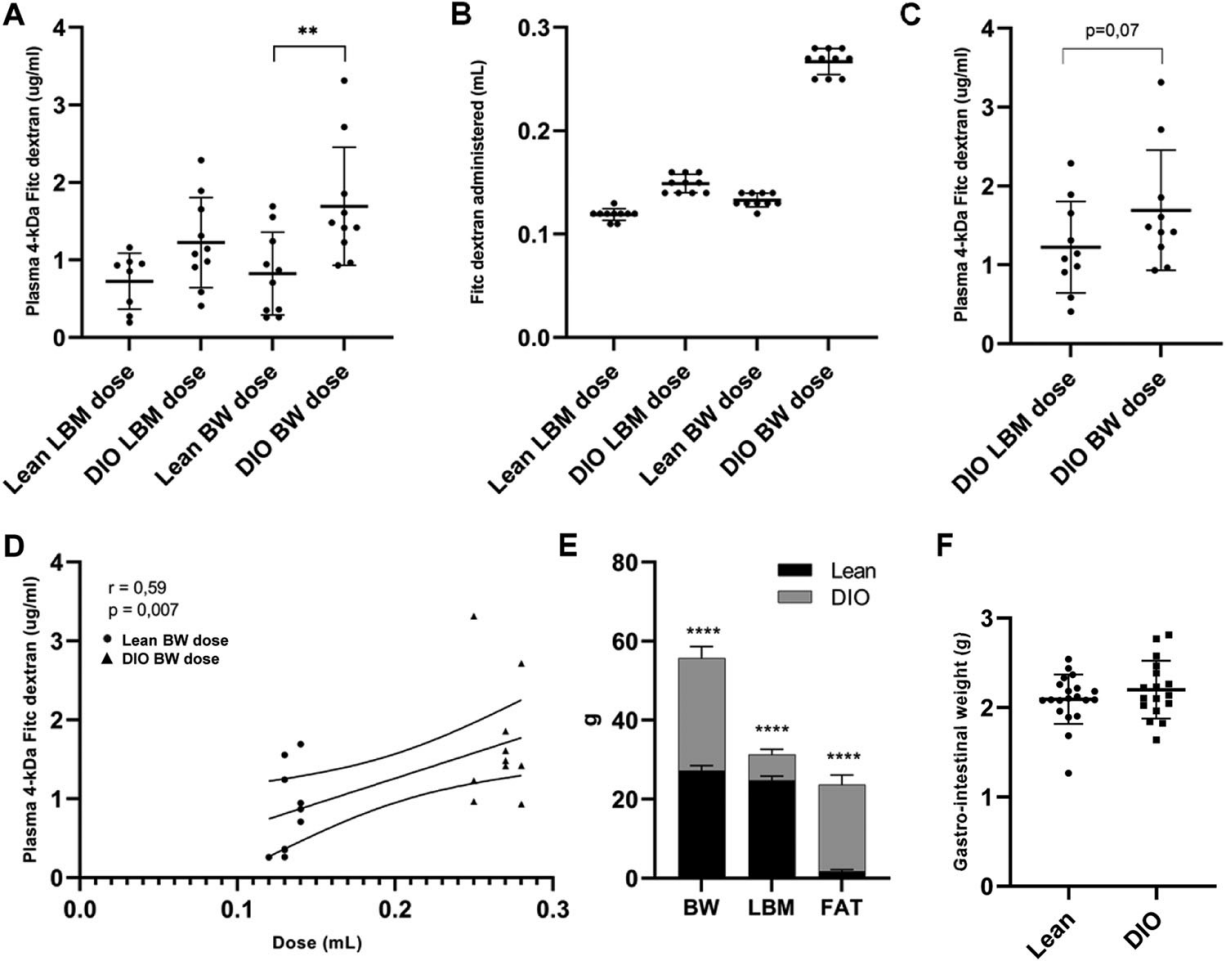
Test of bodyweight-based and lean body mass-based dose regimen in the FITC dextran 4kDA intestinal permeability test. **A** C57BL/6 J mice, on a low-fat diet (Lean) or high-fat diet (DIO) for 18–24 weeks were dosed with FITC dextran 4 kDa (FD-4) (600 mg/kg, n = 10/grp) orally according to lean mass (LBM) or body weight (BW). Two samples were excluded due to hemolysis. **B** The different doses given to the two diet groups according to the assigned dose regime. **C** A close-up of the intestinal permeation of FD-4 seen in the same group of DIO mice dosed due to different dose regimes (**A**). **D** Positive correlation between dose given due to body weight and measured FD-4 in plasma. **E** Bodyweight (BW), Lean mass (LBM), and fat measured in the two diet groups LFD (Lean) and HFD (DIO). **F** The gastrointestinal system was weighed in the two diet groups (*n* = 20/grp). **A** One-way ANOVA, (**C**) unpaired two-tailed *t* test, (**D**) Pearson’s correlation test, (**E**) multiple unpaired two-tailed *t* test with Benjamin, Krieger, and Yekutieli FDR approach. *p < 0,05, **p < 0,01, ****p < 0,0001.

In three consecutive studies, we explored the influence of dose regimen using the FD-4 test comparing intestinal permeability between obese and lean mice dosed with FD-4 based on either BW or LBM. The LBM-based dose was included to avoid a possible dose bias due to increased BW and adiposity in DIO mice. There was a significantly increased plasma level of FD-4 in obese DIO mice compared to lean mice in the BW-dosed groups (Fig. 1A). In groups with LBM-dose of FD-4, no significant difference in plasma level of FD-4 was observed, although a tendency towards increased absorption in the DIO mice was seen. This result reflects the different doses given (Fig. 1B). In the DIO mice, a tendency towards increased FD-4 in plasma in the BW group as compared to the LBM group was observed (Fig. 1C). A significant positive correlation between dose and FD-4 in plasma was only observed in the subgroups dosed according to BW (Fig. 1D).

Body, liver, and gastrointestinal weights were measured in all mice subjected to the study, and the LBM-dose groups had their fat and LBM calculated by Dexa-scanning (Supplementary Table 3). There was an average of 6.5 g difference in LBM between lean and DIO mice compared to a mean difference of 28.7 g in body weight. The increased fat mass in DIO mice accounted for most of this difference (Fig. 1E). The intestines, including the cecum, were weighed, and no significant differences were observed between the two diet groups (Fig. 1F). As expected, the DIO mice showed an increase in liver weight (Supplementary Table 3).

### Serum FITC dextran 4kDa concentrations are dose-dependent

**Fig. 2:**
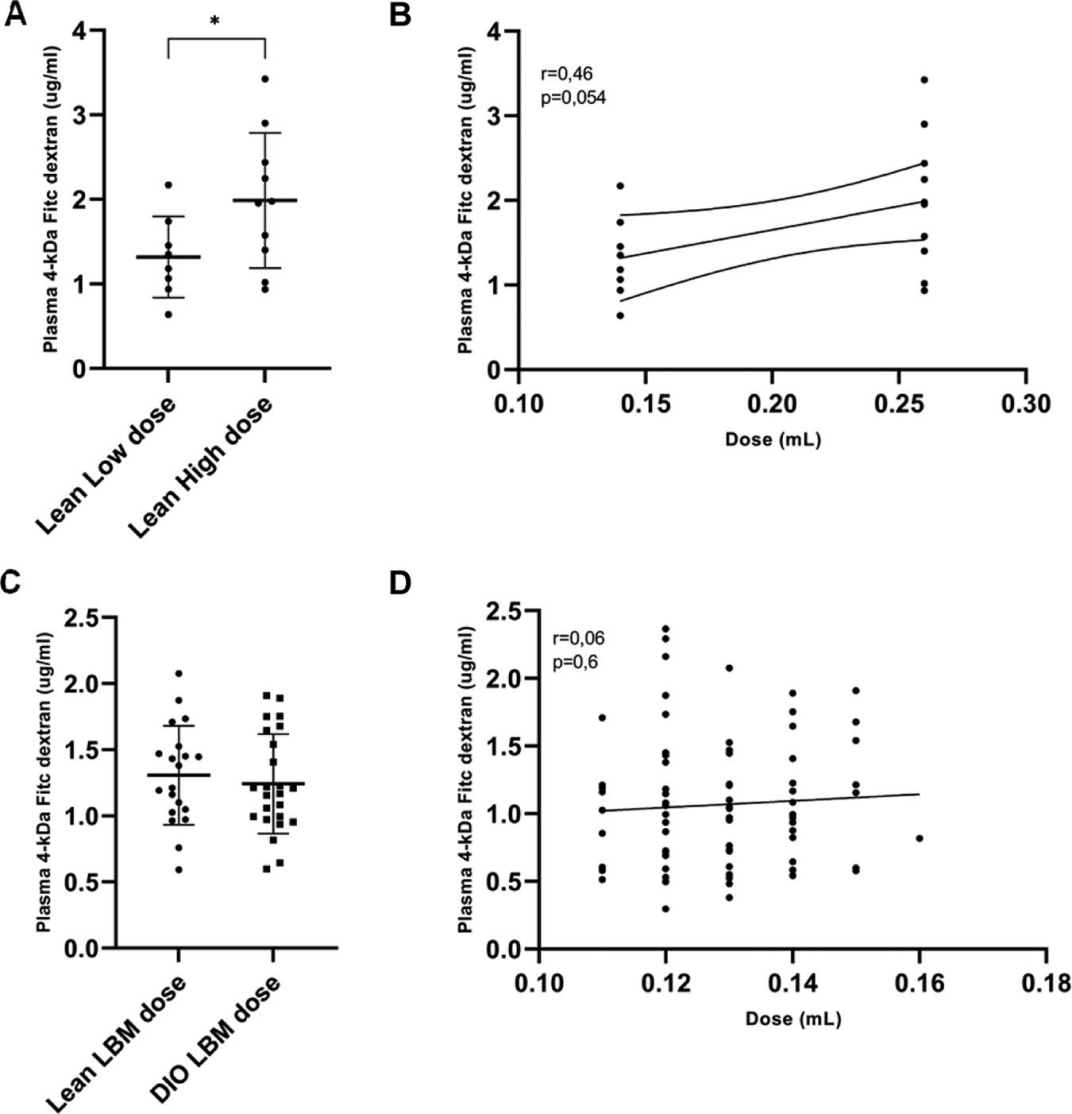
FITC dextran 4kDA intestinal permeability test. **A** C57BL/6 J mice on a low-fat diet (LFD) were weight stratified in two groups, and FITC dextran 4 kDa (FD-4) were dosed PO. in a low dose or high dose, respectively (n = 10/grp). 2 samples were excluded due to hemolysis. **B** Positive correlation between dose given and measured FD-4 in plasma. **C** C57BL/6 J mice on LFD (Lean) or HFD (DIO) (n = 25/grp) were dosed with FD-4 kDa (600 mg/kg) due to measured lean mass. 4 samples were excluded due to hemolysis. **D** No correlation was observed between the dose given due to lean mass and FD-4 measured in plasma. **A** unpaired two-tailed *t* test, (**B**) Pearson’s correlation, (**C**) unpaired two-tailed *t* test, (**D**) Pearson’s correlation. *p < 0,05.

To test the dose-dependency observed in our first study, we dosed weight stratified lean mice with a dose corresponding to either the BW-dose given in lean mice (Low) or obese mice (High). A significant difference was observed between the two dose groups (Fig. 2A). There was a significant positive linear correlation between dose and FD-4 measured in plasma (Fig. 2B).

### Increased intestinal permeability in obese mice is only seen in mice dosed according to body weight

**Fig. 3:**
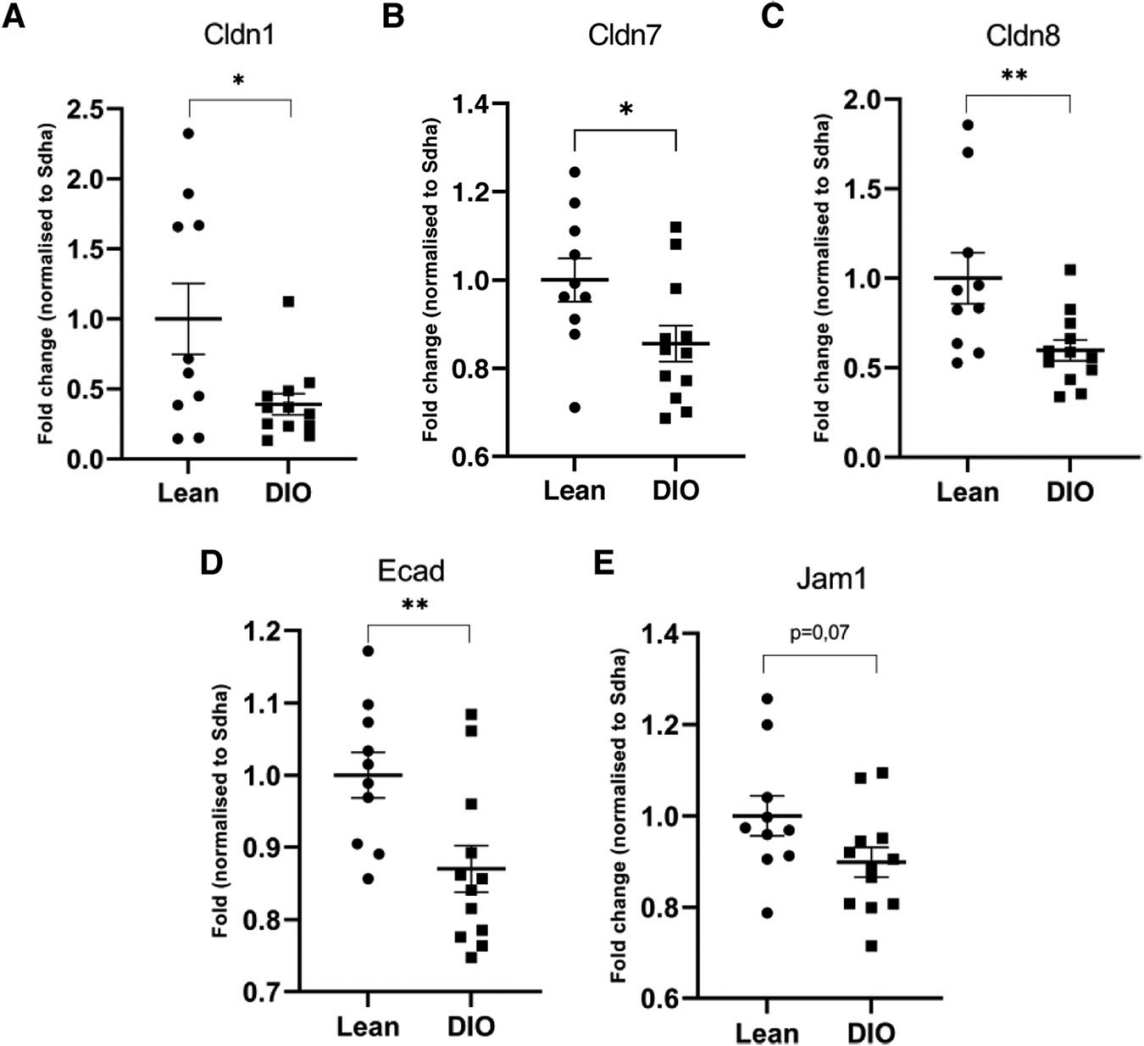
Tight junction and adhesion protein gene expression in the lean and obese mice. The jejunum was sampled from the two diet groups, lean mice on a low-fat diet (LFD) and obese mice on a high-fat diet (DIO) (*n* = 10–12/grp). qPCR analysis was performed for selected tight junctions’ genes (**A**) Claudin 1, (**B**) Claudin 7, (**C**) Claudin 8, (**D**) E-cadherin, and (**E**) Junctional adhesion molecule and normalized to succinate dehydrogenase complex (Sdha). Unpaired two-tailed *t* test. *p < 0.05, **p < 0.001.

To test whether the tendency towards increased intestinal permeability in the LBM dosed subgroups observed in the first study (Fig. 1A) was a true difference, we conducted a new study with larger groups determined by a power calculation (see EMS Table 1). No difference in intestinal permeation of FD-4 was observed between lean and DIO mice (Fig. 2C). As in the first study, there was no correlation between LBM-dose and the FD-4 measured (Fig. 2D). To further access the intestinal barrier in our mouse model, we used qPCR analysis of selected tight junctions’ genes and found that cldn1, cldn7, cldn8, Ecad, and Jam1 were decreased in the DIO mice compared to the lean mice (Fig. 3).

## Discussion

The current studies showed that dose regimen matters when testing intestinal permeability by FD-4 in a DIO model. We showed that the readout is biased by significant differences in dose when comparing lean and obese mice after dosing according to body weight. This can, however, be avoided by dosing according to the LBM. In our studies a mean overestimation of 38% was observed when dosing due to body weight compared to the LBM-based dose regimen.

The FD-4 method utilizes that simple sugars passively move from the gut to the circulation without being metabolized or degraded. The amount passing over the intestinal barrier depends on the barrier’s concentration gradient, surface area, mucosal blood flow, transit time, and intestinal permeability [3, 7]. To determine intestinal permeability, the other factors must be constant. We did not observe differences in intestinal weight measured, suggesting that lean and obese mice have the same surface area. However, it should be noted that an increased villus length and intestinal weight in obese mice may result in a larger surface [10]. Jorgensen and colleagues also found that blood flow in lean and obese mice was comparable [11]. As the gut surface area and blood flow thus seem to be comparable, we conclude that using high BW-based doses for DIO mice that are heavier due to increased adiposity can be misleading.

There is a discrepancy in studies evaluating transit time changes due to HFD, i.e., some studies report similar time, others report increased proximal transit time, and delayed colonic transit time in HFD mice [9, 12]. This highlights the fact that permeability differs in the gut segments, and therefore, attention must be paid to the sampling time reflecting different parts of the intestine. Furthermore, we cannot exclude that differences in volume administered by BW dose regimen might affect gastric emptying rate and hence time of peak of plasma FD-4, but using the LBM dose regimen secure similar volume of FD-4 across study groups.

We found no difference in intestinal permeation when FD-4 was dosed due to LBM, despite an upscale of the group size to account for the considerable variability of the permeability marker used. Nevertheless, the qPCR analysis showed changes in tight junctions guarding the paracellular gut permeability in accordance with previous findings [4–6], which indicate that additional measures are essential for studies evaluating the intestinal barrier due to the limited sensitivity of the FD-4 intestinal permeability test. However, one could argue that if there is a minor change in intestinal permeability, a higher dose is needed to detect this difference.

On the other hand, we have shown that increasing the dose results in an increased absorption to the blood of FD-4 independent of the gut permeability. The shortcomings of the FD-4 intestinal permeability test addressed in our studies have also previously been reported [8]. These studies collectively highlight that dosing according to BW in permeability test is not always linear and emphasise the need for more awareness when applying the in vivo intestinal permeability tests. We recommend that LBM-based doses should be used for the FD-4 intestinal permeability test when comparing lean and obese mice or using a method with standardized doses.

### Study importance

#### What is already known about this subject?

- FD-4 is a preclinical, commonly used, and simple in vivo intestinal permeability test.

#### What is the key question?

- Should obese animals be dosed according to their body weight or their lean body mass, and would it make a difference?

#### What are the new findings?

- The FD-4 intestinal permeation is dose-dependent when using a bodyweight-based dose regimen
- Dosing FD-4 due to lean body mass shows no difference in intestinal permeability between lean and high fat-fed obese mice

#### How might this impact clinical practice in the foreseeable future?

- A more advisedly use of intestinal permeability test, accounting for the properties of the marker molecule and physical parameters affecting the test, in obese mice models will improve the validity of future preclinical studies

## Supplementary information

Supplementary Material

## Data Availability

Data reported in this article is deposited and are public available at 10.17605/OSF.IO/UVBGD.
