# Supplementary material for: The intestinal permeability marker FITC-dextran 4kDa should be dosed according to lean body mass in obese mice
Source: Nutr Diabetes. 2023 Jan 5;13:1. doi: 10.1038/s41387-022-00230-2 (PMC9816099; doi:10.1038/s41387-022-00230-2)
Supplement: Supplementary file 1 — Supplementary Material [file 41387_2022_230_MOESM1_ESM.pdf]

Supplemental Material

Mean 1	Mean 2	SD	Enrolment ratio	Alpha	Beta	Sample size
1.23	0.73	0.58	1	0.05	0.2	21/grp

Table 1 power calculation*. To calculate appropriate sample size to test for increased permeability in high fat fed mice using the lean-body-mass regimen, we used a power calculation based on parameters from the first study [1].*

[1] Rosner B (2011) Fundamentals of biostatistics. Seventh edition. Boston : Brooks/Cole, Cengage Learning, [2011] ©2011

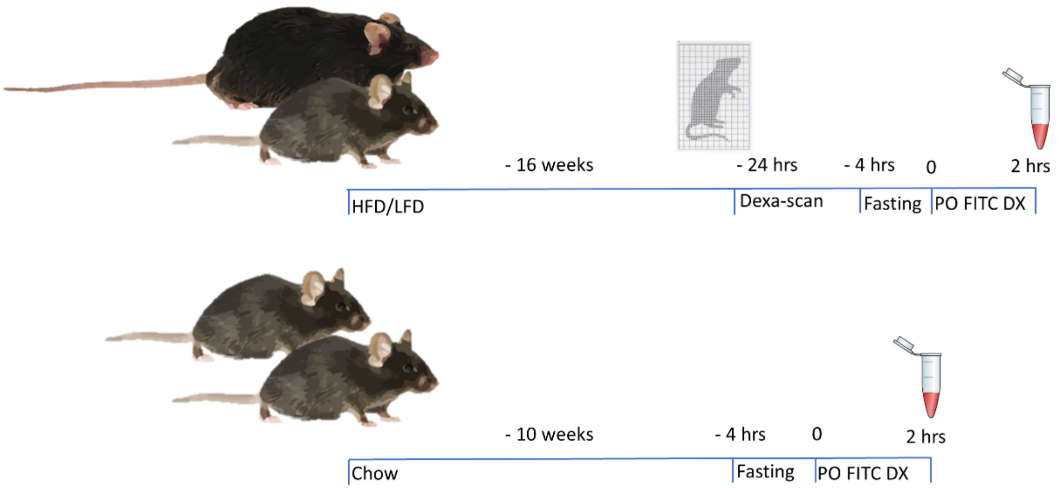

Figure 1 Study design with time indications. *The upper panel illustrates study 1 and the power corrected study. The lower panel illustrates the Dose-response study in lean mice. Mice were fed the assigned diet for 10-16 weeks. On the study day, mice were fasted 4 hours, dosed with FITC dextran 4 kDa PO and blood were sampled 2 hours post-dosing.*

Gene	Assay ID	Gene bank
Cldn 1	Mm00516701_m1	NM_016674.4
Cldn 7	Mm00516817_m1	NM_001193619.1
Cldn 8	Mm00516972_s1	NM_018778.3
E-cad (Cdh1)	Mm01247357_m1	NM_009864.2
Jam-1 (F11r)	Mm00554113_m1	NM_172647.2
*Sdha**	Mm01352366_m1	NM_023281.1

Table 2 TaqMan Assays gene of interest and house-keeping gene*.

Group	Lean LM dose	Lean BW dose	DIO LM dose	DIO BW dose
Body weight (g)	27.2 ± 1.1 (25.5 - 28.5)	27.3 ± 1.5 (24 - 29.1)	55.9 ± 3.4 (51.7 – 62.2)	55.5 ± 2.5 (51.5 - 58.1)
Lean mass (g)	24.8 ± 1.1 (23.2 – 26.2)	-	31.3 ± 1.4 (29.6-33.2)	-
Fat mass (g)	1.7 ± 0.5 (1.1 – 2.4)	-	23.7 ± 2.4 (21 – 29.1)	-
Gastro-intestinal weight (g)	2.2 ± 0.2 (2.1 – 2.5)	2 ± 0.3 (1.3 – 2.3)	2.1 ± 0.2 (1.8 – 2.4)	2.3 ± 0.4 (1.6 – 2.8)
Liver weight (g)	1.2 ± 0.1 (1.01 – 1.3)	1.2 ± 0.2 (0.7 – 1.4)	3.3 ± 1.1 (1.5 – 4.7)	3.6 ± 0.35 (3.1 – 4.2)
Liver weight normalized to body weight (%)	4.5	4.3	6	6.5

Table 3 Overall measurements in the four groups. *The two diet groups, lean mice on the low-fat diet and DIO mice on the high-fat diet, were divided into two subgroups, each allocated to one of two test dose regimens, dose due to lean mass, LM, and dose due to body weight, BW (n=10/grp). Mice were weighed, and the LM groups were also DEXA-scanned before study initiation. Mice were fasted for 4 hours and FITC dextran 4 kDa were dosed orally due to BW or LM. 2 hours after dosing, a blood sample was collected, and the concentration as a measure for intestinal permeability was calculated. After study termination the weight of the intestines and livers were measured. Data ±SD (min-max), g=gram.*
